# The double-edged impact of Internet use on mental health outcomes among Filipino university students: the mediating role of online social support

**DOI:** 10.3389/fsoc.2023.1132523

**Published:** 2023-04-28

**Authors:** Paolo Miguel T. Abad Santos, Jerome V. Cleofas, Arianne Gail O. Austria, Alejandra Kamiya B. de Guzman, Brianna Angela F. Sarile

**Affiliations:** Department of Sociology and Behavioral Sciences, De La Salle University, Manila, Philippines

**Keywords:** Internet use, mental health, mental wellbeing, social support, students, psychological distress

## Abstract

**Introduction:**

Evidence supports both the positive and negative effects of Internet use on mental health outcomes, but it remains unclear on the role of online social support in this relationship. This study examined the link between daily hours of general Internet use and bidimensional mental health (BMMH) through the pathway of online social support (OSSS).

**Methods:**

Drawing from a sample of 247 Filipino university students, this cross-sectional study tested two simple mediation models that considered mental wellbeing and psychological distress as outcome variables.

**Results:**

Findings show that the total effect of Internet use is positive and negative for mental wellbeing and psychological distress, respectively. Online social support mediated the favorable effects of Internet use on BMMH outcomes. However, the introduction of OSSS as a mediator yielded residual direct effects with opposing signs for both models. The resultant inconsistent mediation in the models signifies the double-edged impact of Internet use on mental health, with favorable effects transmitted through online social support.

**Discussion:**

Findings highlight the importance of online social support as a pathway to harness the positive effects of Internet on mental health. Recommendations to improve online social support for students are discussed herein.

## 1. Introduction

Since the dawn of the twenty-first century, the Internet has become an integral component of human life. Internet-based technologies have become increasingly ubiquitous in various aspects of social life, including education, work, commerce, health, family, community, and governance. Humanity's reliance on the Internet has been further compounded due to the novel coronavirus 2019 (COVID-19) pandemic, when in-person interactions and transactions had to be conducted online. In 2022, 4.95 billion (62.5%) of the total population are connected to the Internet (Kemp, 2022a). Due to the increasing necessity of the Internet in daily living, the United Nations (2016) declared that Internet access is a basic human right. However, despite its utility, the Internet has demonstrated negative consequences on individuals and society, which include concerns over data privacy and security, cybercrimes, widening of socioeconomic divides, physical and mental health, destabilization of social structures and institutions, and other threats to individual and collective wellbeing (Quaglio, 2020).

This study draws its focus on Filipino university students and was conducted during the period of COVID-19 pandemic-induced community quarantine and distance learning in the country. There were a total of 76 million Internet users in the Philippines recorded in January 2022 (Kemp, 2022b). The Internet penetration rate in the college-aged (18–24 years old) population is 86% (Statista, 2022). Kemp (2022b) has indicated an almost 3% increase in new Filipino Internet users from 2021 and 2022, arguably due to the online connectivity demands of the pandemic.

A particular research interest in the fields of digital determinants of health and digital sociology is digital mental health, particularly the influence of the Internet and other Internet-related technologies on mental health outcomes and the pathways where these effects are transmitted. Review evidence suggests that Internet use has both positive and negative outcomes for mental health among higher education students (Rouvinen et al., 2021). Local evidence suggests that increased use of particular Internet technologies (i.e., social media) among Filipino undergraduates is associated with mental health changes during the pandemic (Cleofas et al., 2022a). This study also found that whether college students demonstrate good or poor mental health is based on their problematic or reflective use of the Internet. Education-based research in the Philippines has also noted that poor digital access and tools were linked to poor academic and wellbeing outcomes among students (e.g., Cho et al., 2021).

However, research on the role of seeking and receiving social support online on mental health outcomes remains to be unclear, as suggested by Chang et al. (2022). The Philippines is often described as a collectivist culture (Flaming et al., 2010); hence, we argue that online social support is a potential salient mediator between Internet use and mental health, especially during the time of the pandemic, when Filipino students have become more reliant on Internet technologies to meet their social and intimacy needs due to COVID-induced physical distancing (Cleofas et al., 2022b). In this study, online social support is characterized as the experiences of students in socializing online in order to access benefits and fulfill their emotional, informational, companionship, and instrumental needs. These facets of social support in the online space has been indicated as promoters of mental health. Hence, this study attempts to determine the mediating role of online support on the relationship between Internet use and mental health outcomes.

## 2. Literature review and conceptual framework

### 2.1. Internet use and wellbeing theory

This study appeals to the theoretical framework of Castellacci and Tveito (2018) that suggests explanatory pathways wherein Internet use can improve wellbeing outcomes. They posit that Internet use exposes individuals to opportunities to improve the personal and work lives of individuals. The positive effects of Internet usage on the various domains of life can improve the culture, capabilities, and psychological functioning, which in turn optimizes wellbeing. This is confirmed by a review of higher education studies, which suggests that Internet use can promote physical, mental, social, and intellectual health among university students (Rouvinen et al., 2021). Moreover, Castellacci and Tveito's (2018) proposed that the Internet facilitates positive life outcomes by introducing changes in the overall use of time. Hence, the independent variable of interest for this study is daily hours of Internet use (DHIU), simply assessed through the self-reported average number of hours of Internet usage per day.

### 2.2. Bidimensional mental health

Castellacci and Tveito (2018) Internet use and wellbeing theory proposed that Internet use can enhance psychological functioning (i.e., mental health). Moreover, Rouvinen et al. (2021) suggested that Internet usage can influence the mental health of higher education students. The present study conceptualizes mental health using the bidimensional model (Renshaw and Bolognino, 2017). The bidimensional model (or dual-factor) of mental health (BMMH) explains that the assessment of mental health outcomes must be two-pronged and should include both positive and negative aspects of mental health. This study operationalizes the positive component of BMMH as ***mental wellbeing*** (Tennant et al., 2007), which refers to a eudemonic and hedonistic state where one feels optimistic, useful, relaxed, close to people, and able to think clearly, decide, and deal with one's problems. On the other hand, the negative component of BMMH is operationalized as ***psychological distress*** (Kessler et al., 2003), which refers to the extent one feels anxious, depressed, nervous, and restless. Mental wellbeing and psychological distress are the two dependent variables of interest in the present study.

On the other hand, in terms of the relationship between Internet use and mental health outcomes, results from the extant literature vary based on the measures used for the variables and the type/specificity of the Internet technology used (e.g., social media and Internet gaming). Rouvinen et al. (2021) suggest that Internet use in higher education can lead to promotive and/or detrimental effects on mental health. On the one hand, prior research among university students has evinced the link of various Internet-related technologies to positive mental health outcomes, such as higher mental wellbeing (Cleofas et al., 2022a), attainment of psychosocial developmental tasks (Cleofas et al., 2022b), satisfaction with daily habits (Austin-McCain, 2017), improved stress management (Saini et al., 2020), enhanced resilience (Sage et al., 2021), and engagement in psychological interventions (Lattie et al., 2019). On the other hand, findings from systematic reviews of studies among university students have demonstrated the association of Internet use with poor mental health outcomes, such as depression, anxiety, maladaptive cognitions, behavioral addictions, and loneliness (Rouvinen et al., 2021; Valkenburg et al., 2022). Hence, our hypotheses for the relationship between Internet use and mental health are non-directional:

H_1a_: Daily hours of Internet use predicts mental wellbeing.
H_1b_: Daily hours of Internet use predicts psychological distress.

### 2.3. Online social support

The theory of Castellacci and Tveito (2018) explains that the nature of the influence of Internet use on psychological functioning (i.e., mental health) is based on particular life domains where the Internet is used by an individual. Among the four domains identified by Castellacci and Tveito, the present study will focus specifically on ***social life***. For this study, the social domain of Internet use is operationalized as ***online social*** ***support*** (OSSS). Following Nick et al. (2018), OSSS is constituted of four types of social support received or experienced by individuals in an online environment. These include esteem and emotional support (e.g., receiving validation and care online), social companionship (e.g., experiencing a sense of belongingness online), informational support (e.g., sharing new information or perspectives and solving problems online), and instrumental support (e.g., receiving material support or services online). Online social support can exist in any online platform (Nick et al., 2018); hence our use of a generalized measure of Internet use as hours per day.

In particular, during the period of the COVID-19 pandemic when this study was conducted, research in the Philippines (Cleofas et al., 2022b) and elsewhere (e.g., Onat Kocabiyik, 2021; Bahfiarti and Arianto, 2022) have demonstrated the need for social connections and support as primary motivations for students to increase their use of Internet-based technologies. Previous research has indicated that those who spend more time online receive higher levels of online social support (Nick et al., 2018). Thus, we hypothesize that:

H_2_: Daily hours of Internet use positively predicts online social support.

Furthermore, online social support has also been shown to have beneficial effects on mental health. For instance, Nick et al. (2018) have indicated that OSSS increases self-esteem and decreases depressive symptoms. In addition, Ali (2020) suggests that OSSS is a positive predictor of psychological wellbeing. Evidence from a systematic review has linked online social support with improved satisfaction with life and self-identity, and decreased social anxiety and loneliness (Zhou and Cheng, 2022a). Taken together, these studies indicate that OSSS is a facilitating factor of mental wellbeing and a protective factor against distress. Hence, we hypothesize that:

H_3a_: Online social support positively predicts mental wellbeing.
H_3b_: Online social support negatively predicts psychological distress.

Moreover, Castellacci and Tveito's (2018) also proposed that in terms of its relationship to psychological functioning and wellbeing, the Internet provides both opportunities to flourish and risks to flounder. This proposition is further evinced by the previous section that demonstrates the double-edged effect of Internet use on mental health outcomes. Castellacci and Tveito further posit that the social-domain-related function of the Internet can be examined as a channel through which the positive or negative impact of the Internet on psychological functioning (i.e., mental health) can be transmitted. This mediating role of social use of Internet is evinced by prior studies (Brailovskaia et al., 2021; Meshi and Ellithorpe, 2021; Chang et al., 2022). Thus, there is reason to suspect that online social support is a social domain that can mediate and further explain the relationship between Internet use and the BMMH outcomes among college students.

H_4a_: Daily hours of Internet use has an indirect effect on mental wellbeing through the pathway of online social support.
H_4b_: Daily hours of Internet use has an indirect effect on psychological distress through the pathway of online social support.

### 2.4. The present study

**Figure 1:**
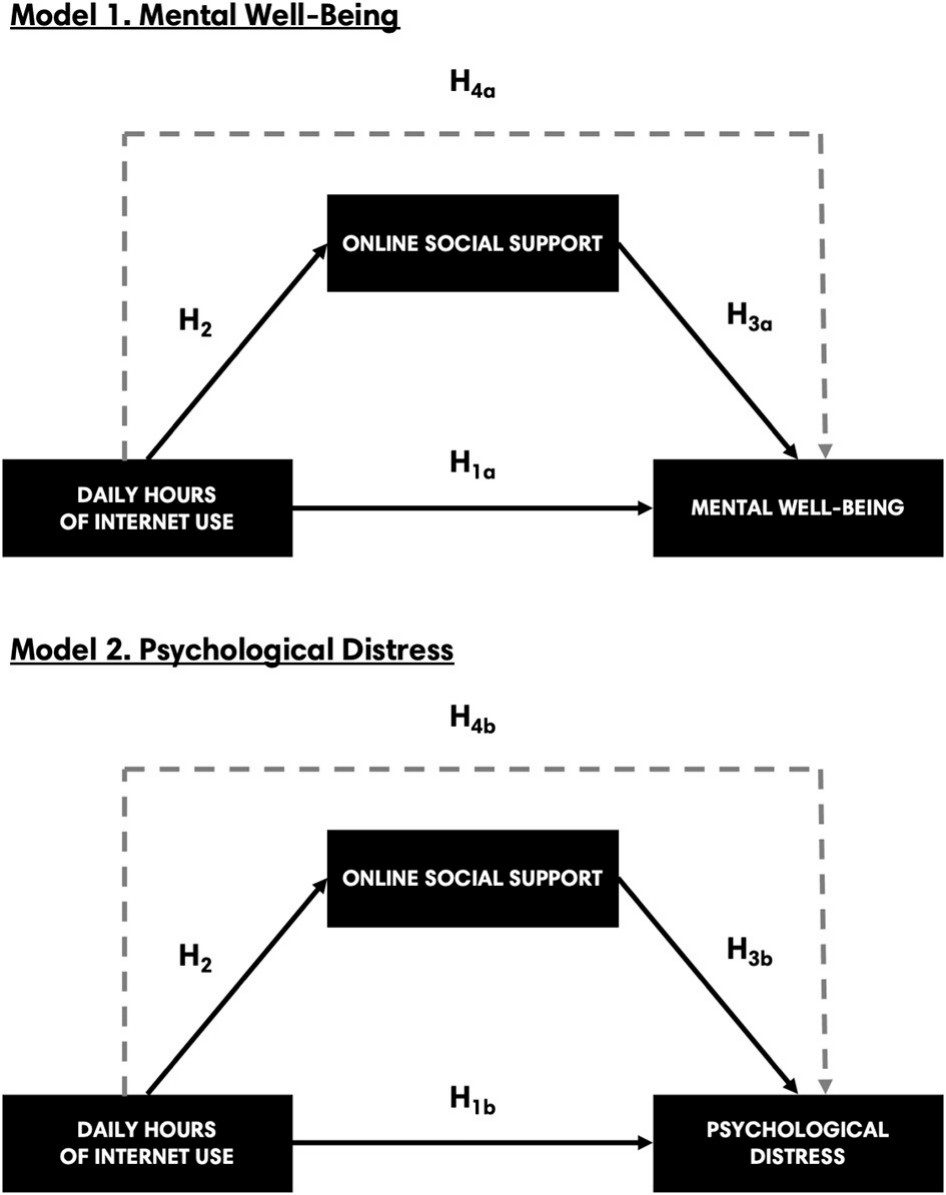
Two simple mediation models: Model 1 presents the hypothesized indirect effect of DHIU on mental wellbeing through OSSS; Model 2 presents the hypothesized indirect effect of DHI on psychological distress through OSSS.

The aim of the present study is to examine the relationship between daily hours of Internet use and BMMH outcomes and the mediating role of online social support in this relationship among Filipino university students. Cognizant of the current state of literature that indicates the inconsistent association between general Internet use and mental health, we attempt to contribute by offering an explanatory pathway by including OSSS as a mediator, following the theoretical assertions of Castellacci and Tveito's (2018). Two simple mediation models representing the two BMMH outcomes (i.e., mental wellbeing and psychological distress) will be tested to address the hypotheses of the study (see Figure 1). While there had been studies in the past that tested similar models, they focused on offline or generalized social support (Glaser et al., 2018; Lin et al., 2021) and/or did not use BMMH as a framework to measure mental health as outcomes (Brailovskaia et al., 2021; Meshi and Ellithorpe, 2021; Chang et al., 2022). Additionally, these studies had inconsistent findings. We believe that specifying the type of social support (i.e., online) and assessing dual-factor mental health outcomes can help in drawing more nuanced insights about the variables and relationships being tested.

## 3. Methods

### 3.1. Research design and participants

This study utilized a quantitative, cross-sectional research design, specifically simple mediation analysis. A simple mediation study examines how the effect of an independent variable (i.e., Internet use) on a dependent/outcome variable (i.e., mental health outcome) is transmitted through a mediating variable (i.e., online social support) (Fritz and Mackinnon, 2007). The target participants for this study are undergraduate students from a selected private university in Manila, Philippines. The respondents were recruited regardless of their degree, college, or duration of curriculum. This is to ensure that all sectors of the student community are represented in the study. Following Fritz and Mackinnon (2007), the minimum sample size required to achieve 0.80 power for one simple mediation model using the variables in this study is 88 (α = 0.39, β = 0.59, τ′ = 0.39). Hence, two mediation models will require at least 176 respondents. The final sample size of the study is 247. A plurality of the respondents are 21 years old (25.1%, *M* = 20.06, *SD* = 1.40). Males (51%) and females (49%) are approximately equal in representation.

### 3.2. Study procedure and ethical considerations

The data for this study was collected via an online survey (Google Forms) during the first quarter of 2022. Convenience sampling was employed to recruit respondents. The link was sent to the email addresses and social media accounts of the target participants. The study protocol is adherent to the principles of the Declaration of Helsinki and was granted administrative clearance for ethical conduct of research from the researchers' home department at the university. Informed consent was secured from all respondents through the first page of the online survey form. No private and personal details were collected from the participants. All data gathered were anonymized and secured in a double authenticated cloud storage.

### 3.3. Instruments

#### 3.3.1. Daily hours of internet use

To measure self-reported DHIU, we asked, “on average, how many hours do you spend on the Internet daily?” on the survey. They were instructed to type in a number from 0 to 24 in the textbox provided with the question.

#### 3.3.2. Online social support scale

OSSS is a 40-item scale developed by Nick et al. (2018) to measure the extent to which respondents receive social support in the forms of emotional, social companionship, informational and instrumental while they interacted with others on online platforms over the last 2 months. For each item, respondents were asked how often they experienced particular social interactions (e.g., “*When I'm online, I talk or do things with other people*”) and were instructed to respond using a 5-point scale (0 = never, 4 = a lot). The OSSS exhibited high internal consistency (α = 0.94–0.95) and strong psychometric properties (see Nick et al., 2018). Scores ranged from 0 to 160. For this sample, the Cronbach alpha score for OSSS is 0.992. According to the developers' website, the use of the OSSS is free as long as attribution and citation of the authors are made.

#### 3.3.3. Short Warwick-Edinburgh mental wellbeing scale

SWEMWBS is a 7-item scale that assesses positive aspects of mental health (Tennant et al., 2007). Participants were instructed to respond to each item that described experiences of mental wellbeing (e.g., “*I've been able to make up my own mind about things*.”) in the last 2 weeks using a 5-point scale (1 = none of the time, 5 = all of the time). Scores were transformed based on developer instructions. SWEMWBS has demonstrated robust psychometric properties (e.g., Ringdal et al., 2018), including an acceptable reliability score in the Philippine population (α = 0.87, Cleofas and Oducado, 2022). The range of possible scores is from 7 to 35. For this sample, SWEMWBS garnered a Cronbach alpha value of 0.962. The use of this instrument has been registered at the developer's website.

#### 3.3.4. Kessler psychological distress scale

K10 is a 10-item brief assessment for psychological distress developed by Kessler et al. (2003). K10 is a widely used scale to estimate the likelihood of mental health problems, such as depression, anxiety, stress, and agitation. The respondent was asked to rate each item (e.g., “*In the past 4 weeks, about how often did you feel restless or fidgety*?”) using a 5-point scale (1 = none of the time, 5 = all of the time). K10 has been noted to have good internal consistency (α = 0.84) and construct validity (see Hoffman et al., 2022). Scores ranged from 10 to 50. For this sample, the Cronbach Alpha value for K10 is 0.968. Permission was sought for the use of this instrument.

### 3.4. Data analysis procedure

Mean and standard deviation was used to summarize the key variables. Bivariate correlations among the variables were tested using Pearson r correlation. Mediation analyses, including the computation of total, indirect and direct effects and path estimates, were carried out using the Mediation Module of JAMOVI version 2.0.0.0. Kolmogorov-Smirnov test results for the key variables signify normality. Bootstrapping using 5,000 replicates was performed in the mediation. *R*^2^ scores for OSSS, SWEMWBS, and K10 were computed using the Mediation Analysis module of JASP 0.16.1. Significance level was set at *p* < 0.05.

## 4. Results

### 4.1. Descriptive and bivariate statistics

Descriptive results in Table 1 indicate that college youth respondents spend an average of 10.312 h (*SD* = 5.120) on the Internet daily. Daily hours of Internet use (DHIU) ranged from 2 to 24 h. On the other hand, the mean score for online social support (OSSS) is 98.457 (*SD* = 43.325, Range = 16–160). As for mental health outcomes, average SWEMWBS scores indicate that respondents experience moderate mental wellbeing (*M* = 21.112, *SD* = 6.739), while K10 scores indicate that they are likely to have a moderate disorder (*M* = 29.437, *SD* = 12.118).

**Table 1:** Descriptive statistics, internal consistency scores, and correlational analysis among key variables (*N* = 247).

**Variables**	**Mean (SD)**	**Range**	**Cronbach's alpha**	**Correlation coefficients**		
				**(1)**	**(2)**	**(3)**
(1) DHIU	10.312 (5.120)	2–24	–			
(2) OSS	98.457 (43.325)	16–160	0.992	0.395^***^		
(3) SWEMWBS^a^	21.112 (6.739)	7–35	0.962	0.146^*^	0.802^***^	
(4) K10^b^	29.437 (12.118)	10–50	0.968	−0.194^**^	−0.742^***^	−0.866^***^

^*^p < 0.05.

^**^p < 0.01.

^***^p < 0.001.

^a^Interpretation for SWEMWBS: low = 7–20; moderate = 21–27; high = 28–35.

^b^Interpretation for K10: likely to be well = 10–19; likely to have a mild disorder = 20–24; likely to have a moderate disorder = 25–29; likely to have a severe disorder = 30–50.

Bivariate statistical results indicate that DHIU is significantly positively correlated with OSSS (*r* = 0.395, *p* < 0.001). Moreover, SWEMWBS demonstrated significant positive correlations with DHIU (*r* = 0.146, *p* = 0.021) and OSSS (*r* = 0.802, *p* < 0.001). Meanwhile, psychological distress (K10) exhibited significant negative correlations with DHIU (*r* = −0.194, *p* = 0.002) and OSSS (*r* = −0.742, *p* < 0.001) (see Table 1). This means that higher usage of the Internet was observed among those with high online social support and mental wellbeing, and low psychological distress.

### 4.2. Simple mediation analyses results

The study carried out two simple mediation analyses, examining whether the relationship between Internet use and mental health outcomes (i.e., mental wellbeing and psychological distress) would be mediated by online social support. Table 2 presents the direct, indirect, and total effects among the key variables of the study.

**Table 2:** Simple mediation analyses for daily hours of Internet use (DHIU), online social support (OSS), and bidimensional mental health outcomes (i.e., SWEMWBS and K10).

**Paths**	**Estimates (95%CI)** ^**a**^		
	**Direct effect**	**Indirect effect**	**Total effect**
**Model 1 (Mental wellbeing):**			
DHIU → OSS	3.345 (2.425 to 4.276)^***^		
OSS → SWEMWBS	0.137 (0.126 to 0.149)^***^		
DHIU → SWEMWBS	−0.267 (−0.374 to −0.167)^***^		0.192 (0.031 to 0.330)^**^
DHIU → OSS → SWEMWBS		0.459 (0.325 to 0.598)^***^	
**Model 2 (Psychological distress):**			
DHIU → OSS	3.345 (2.377 to 4.191)^***^		
OSS → K10	−0.220 (−0.2416 to −0.199)^***^		
DHIU → K10	0.277 (0.046 to 0.515)^*^		−0.460 (−0.716 to −0.160)^**^
DHIU → OSS → K10		−0.737 (−0.966 to −0.516)***	

^*^p < 0.05.

^**^p < 0.01.

^***^p < 0.001.

^a^Bootstrapped estimates using 5,000 replicates.

Model 1 (mental wellbeing model) results suggest that without accounting for OSSS, the total effect of DHIU on SWEMWBS is significant and positive in nature (*B* = 0.192, *p* = 0.009). When we added OSSS as a mediating variable, the indirect effect of DHIU on SWEMWBS was significant and positive in nature (*B* = 0.459, *p* < 0.001); however, the residual direct effect of DHIU on SWEMWBS, although significant, became negative (*B* = −0.267, *p* < 0.001). The opposing signs of the direct and indirect effects suggest an *inconsistent mediation*. Path estimates indicate that DHIU positively predicts OSSS (*B* = 3.345, *p* < 0.001), and OSSS positively predicts SWEMWBS (*B* = 0.137, *p* < 0.001). Model 1 explains 15.6% of the variance of OSSS and 67.9% for SWEMWBS. This means that the effects of Internet on mental wellbeing may be positive and negative, with the positive effect coursing through online social support.

Model 2 (psychological distress model) results indicate that before accounting for OSSS, the total effect of DHIU to K10 is significant and negative in nature (*B* = −0.460, *p* = 0.001). When we included OSSS as a mediating variable, the indirect effect of DHIU on K10 was significant and negative in nature (*B* = −0.737, *p* < 0.001); however, the resulting residual direct effect of DHIU on K10, albeit significant, became positive (*B* = 0.277, *p* < 0.020). Like the first model, the competing signs of the direct and indirect effects in model 2 also indicate an *inconsistent mediation*. Path estimates suggest that DHIU positively predicts OSSS (*B* = 3.345, *p* < 0.001), and OSSS negatively predicts K10 (*B* = −0.220, *p* < 0.001). Model 2 explains 15.6% of the variance of OSSS and 56.2% for K10. This means that the effects of Internet on psychological distress may be positive and negative, with the negative effect coursing through online social support.

## 5. Discussion

The main objective of this study is to describe the role of online social support as a pathway through which Internet use can influence bidimensional mental health among Filipino students in a selected private university. Our research extends the literature by providing empirical evidence that links social-domain-related use of Internet-based technologies can explain how the Internet facilitates wellbeing. Similar to the outcomes of previous COVID-19 research done among Filipino adults (Egcas et al., 2021; Aruta, 2022; Cleofas et al., 2022a), the findings of the present study indicate that college student respondents have moderate mental wellbeing and are likely to experience moderate psychological distress symptoms. During the time this study was conducted, it was the second year of the pandemic and community quarantine in the Philippines. The sustained restrictions due to COVID-19 may explain the persistence of below optimal mental health of the students. These sustained levels in BMMH outcomes support the claim that the pandemic-induced societal disruptions can cause lasting effects on psychosocial health and developmental outcomes of young people during the latter periods of and even beyond the outbreak (Settersten et al., 2020).

Moreover, our findings confirm our first hypothesis: when unmediated, Internet use has a positive and negative total effect on mental wellbeing and psychological distress, respectively. This is consistent with prior evidence, which indicates the promotive influence of using Internet-based technologies on psychological functioning (Castellacci and Tveito, 2018) and mental health (Rouvinen et al., 2021). Moreover, Internet and gadget access were noted as protective factors against poor mental health among Filipino college students during the early months of the COVID-19 pandemic (Cleofas and Rocha, 2021).

### 5.1. Internet use facilitates online social support

As regards the second hypothesis of the study, our findings indicate a significant positive relationship between DHIU and OSSS. This corroborates the results of previous studies in the United States (Nick et al., 2018; Politte-Corn et al., 2022) and China (Chang et al., 2022). In the Philippines, Internet-based technologies, such as social media, have been used to meet belongingness needs, such as seeking help and receiving care from friends and family members and getting information from classmates among undergraduate students during the period of COVID-19 (Cleofas et al., 2022b).

### 5.2. Online social support is linked to favorable mental health outcomes

Our third set of hypotheses is confirmed: online social support positively and negatively predicts mental wellbeing and psychological distress, respectively. The link between online social support and higher wellbeing outcomes has also been indicated by previous studies in Norway (Brandtzaeg and Lüders, 2021) and China (Zhou and Cheng, 2022b). On the other hand, studies in the United States (Nick et al., 2018; Politte-Corn et al., 2022) and Germany (Brailovskaia et al., 2021) suggested that OSSS is inversely correlated with mental health challenges such as stress, depression, anxiety, and behavioral addictions.

### 5.3. The double-edged impact of DHIU and BMMH outcomes when mediated by OSSS

For both the SWEMWBS and K10 models, two key findings emerged when OSSS was accounted for as a mediator between DHIU and BMMH outcomes. First is that online social support significantly mediates the relationship between Internet use and mental health, such that DHIU's positive effect on mental wellbeing and negative effect on psychological distress is strengthened when transmitted through OSSS. This finding confirms Castellacci and Tveito's (2018) proposition: the Internet affords individuals opportunities for social connections and community building, which improves their access to social support and, consequently, improves psychological functioning. Access to the Internet exposes university students to engage in socializing, expand social capital, academic support from other university entities, and receive informational support for health needs (Rouvinen et al., 2021; Cleofas et al., 2022b). These online-based social benefits of the Internet help promote mental wellness and decrease psychopathologies, as seen in prior research (Castellacci and Tveito, 2018; Nick et al., 2018), especially during the social distancing periods of the pandemic when most social interactions transitioned online (Brandtzaeg and Lüders, 2021; Cleofas et al., 2022a).

Second, after the inclusion of online social support as a mediator, estimates of the direct effect of DHIU on BMMH outcomes yielded signs that oppose the total and indirect effect estimates. On the one hand, Internet use transmitted through the channel of online social support increases mental wellbeing and decreases psychological distress. On the other hand, the unmediated residual direct pathway reveals detrimental effects of DHIU on BMMH outcomes. Similarly, studies have demonstrated various socio-behavioral variables as inconsistent or competing mediators of Internet technologies and psychological outcomes (Chan, 2014; Zeng et al., 2021; Chang et al., 2022; She et al., 2023). This inconsistent mediation signifies the double-edged impact of Internet use on mental health and supports the theoretical claims of the Internet use and wellbeing theory, wherein Internet-based technologies can have both positive and negative effects on wellbeing based on how they are used, and the personal and social characteristics of individuals that use them (Castellacci and Tveito, 2018). This two-pronged nature of Internet-social/mental health relationships has also been noted in the university student population (Rouvinen et al., 2021).

### 5.4. Limitations

Our study has several limitations. First, ours is a non-random sample from a single university and culture, which may decrease the generalizability of our results. Second, we only used a regression-based strategy to test our mediation model instead of more robust statistical methods like structural equation modeling (SEM). Moreover, our study could have benefited from a more scale-based measure of Internet use instead of the simple self-reported number of hours of Internet use daily. Additional variables like social media use could not be included due to limitations of sample size. Future studies should consider more specific measures of Internet use, administer to a larger random non-student sample, include covariates and other mediating variables, and compute using more advanced statistical tools. Lastly, since this study is cross-sectional in nature, causality among the relationships could not be determined. Future research should design longitudinal protocols further examine the model forwarded in this study.

## 6. Conclusion

In conclusion, the findings of the present study indicate the double-edged impact of Internet use on bidimensional mental health among Filipino university students, and the role of online social support as a pathway that affords the benefits of the Internet on mental health. Internet use contributes to enhanced mental wellbeing and decreased psychological distress through increased online social support.

### 6.1. Implications

Theoretically, the present study contributes to the field of digital mental health by describing the opposing effects of Internet use on mental health outcomes and how its favorable impact on mental health is transmitted through online social support in the case of Filipino university students. The study also provided empirical evidence that demonstrates selected theoretical claims of the Internet use and wellbeing theory (Castellacci and Tveito, 2018), particularly the effect of Internet usage on psychological functioning through the channel of social life. Future research can further utilize the other life domains proposed by the theory, such as work and consumption, and other personal (i.e., capabilities and culture) and environmental factors (i.e., physical and social-institutional) that may help in explaining the pathway leading to negative effects on Internet on BMMH outcomes.

As regards practical implications, our study provides evidence that can inform mental health promotion practitioners in developing in-person and telepsychology interventions that empower students to positively exploit the social-support opportunities found in online platforms. Moreover, school administrators, teachers, and personnel should provide online and offline activities venues for students to socialize, gain social support and increase social capital. We also recommend the creation of programs and policies that can capacitate salient actors in the lives of students (i.e., family, friends, classmates, teachers, health, helping professionals, and community leaders) on how to provide effective social support online.

## Data availability statement

The raw data supporting the conclusions of this article will be made available by the authors, without undue reservation.

## Ethics statement

The studies involving human participants were reviewed and approved by Department of Sociology and Behavioral Sciences, De La Salle University Manila. The patients/participants provided their written informed consent to participate in this study.

## Author contributions

PA, AA, AG, and BS: conceptualization, protocol development, data gathering, initial data analysis, and writing of initial draft. JC: conceptualization, protocol development, final data analysis, and writing of final manuscript. All authors contributed to the article and approved the submitted version.
